# Self-assessment of learning outcomes in prehospital disaster response skills: instrument development and validation for mass casualty incident training

**DOI:** 10.1136/bmjopen-2024-098284

**Published:** 2025-03-27

**Authors:** Fredrik Schulz, Magnus Hultin, Lina Gyllencreutz

**Affiliations:** 1 Department of Nursing, Umeå University, Umeå, Sweden; 2 Department of Diagnostics and Intervention, Umeå University, Umeå, Sweden

**Keywords:** Decision Making, ACCIDENT & EMERGENCY MEDICINE, MEDICAL EDUCATION & TRAINING, Triage

## Abstract

**Abstract:**

**Objective:**

Measuring the effectiveness of mass casualty incident (MCI) scenario training is challenging due to simultaneously assessing individual skills, team dynamics, decision-making under pressure and adaptability. Existing instruments often focus too narrowly on individual skills, overlooking the comprehensive range of skills needed for effective prehospital disaster response. This study aims to develop and validate a comprehensive self-assessment tool for prehospital disaster response skills during initial MCI scenario training.

**Design:**

The instrument was developed and validated using a comprehensive methodology. This included literature reviews to identify the construct, ensuring content validity through expert evaluation and conducting field trials in MCI scenario training to evaluate the instrument under simulated conditions that approximated real-life incidents. The instrument’s psychometric properties were assessed using exploratory factor analysis (EFA) and Horn’s parallel analysis, as well as Cronbach’s α and item–total correlation analysis.

**Setting:**

Two field trials conducted with participants in Sweden during 2023 and 2024.

**Participants:**

75 students from a bachelor’s programme at a Swedish university were recruited to participate in the field trials. The programme featured one semester of comprehensive theoretical and practical training in disaster medicine, including MCI response and management. 88 instruments were collected during the field trials.

**Results:**

Overall Cronbach’s α score was 0.86, indicating high internal consistency for the instrument. EFA and Horn’s parallel analysis revealed a five-factor model accounting for 52.3% of the total variance: *incident control and management; systematic examination procedures; risk assessment and management; stress response and impact*; and *triage procedures*. Cronbach’s α for all factors indicated good internal consistency (range: 0.74–0.85).

**Conclusions:**

The instrument addresses a critical gap by offering a comprehensive self-evaluation tool for disaster response skills. The robust psychometric properties indicate its potential for practical implication. Future studies should explore its application in diverse training settings and populations to enhance its utility and generalisability.

A comprehensive development and validation methodology ensured the high content validity of the instrument.

**STRENGTHS AND LIMITATIONS OF THIS STUDY**

- A comprehensive development and validation methodology ensured the high content validity of the instrument.
- The instrument addresses a critical gap by evaluating a wide range of the skills required for effective mass casualty incident response.
- The study was conducted with a relatively small and homogenous sample of students from a single university, which may limit the generalisability of the findings to other populations and settings.
- Complementing self-assessment with additional performance measures could provide a more comprehensive evaluation of the instrument’s effectiveness.

## Introduction

 The dynamic and often highly volatile nature of mass casualty incidents (MCIs) places a significant demand on emergency medical responders (EMRs) to be well trained and prepared.^1^ In order to succeed and remain safe during an MCI, it is essential for EMRs to possess a deep understanding of the actions to be performed and the corresponding knowledge of how to execute them. Consequently, it is vital to use training methods that are suitable for practising fundamental skills and competencies.^2^ These training methods must encompass both technical and non-technical skills, as EMRs are required to navigate the multifaceted challenges of providing emergency medical care during an MCI. This includes not only the execution of medical procedures but also effective communication, decision-making under pressure and coordination with other emergency services.^3^

To ensure the effectiveness of these training methods, evaluating the outcomes of training programmes is crucial. Systematic assessment of learning outcomes allows for the identification of strengths and areas for improvement among EMRs.^4^ Furthermore, robust evaluation mechanisms contribute to the development of evidence-based training programmes, ensuring that the skills taught are relevant and effective in real-world scenarios.^5^

Evaluating MCI scenario training presents unique challenges due to the complexity and unpredictability of such events. Unlike routine emergency situations, MCIs involve multiple casualties, diverse injuries, and the need for coordinated responses from other emergency services.^6^ This inherent complexity makes it difficult to design training scenarios that accurately reflect the chaotic nature of actual MCIs.^7^ Additionally, the effectiveness of MCI training is hard to measure because it requires assessing not only individual skills but also team dynamics, decision-making under pressure and the ability to adapt to rapidly changing situations.^1 5^

Numerous evaluation instruments have been developed to target specific skills pertaining to prehospital disaster response, such as situational awareness and teamwork skills. Specifically, the Situation Awareness Global Assessment Technique measures situation awareness at three levels: perception, which is the ability to notice critical elements in the environment; comprehension, which involves understanding the significance of these elements; and projection, which entails predicting future states based on current understanding.^8^ Similarly, the Self-Assessment Teamwork Tool for Students focuses on self-assessment of teamwork skills, including communication, or the ability to effectively exchange information; coordination, the ability to organise team activities; and cooperation, the ability to work collaboratively towards common goals.^9^

While the abovementioned instruments effectively assess individual skill sets, they do not comprehensively evaluate prehospital disaster response skills.^10^ This narrow focus limits the ability to assess the comprehensive range of skills required for effective MCI response.^11^ Consequently, it is crucial that such instruments are further developed and validated to encompass a broader range of the competencies required to manage the initial chaotic phase of an MCI, in order to ensure that training programmes effectively improve the readiness and response capabilities of EMRs. This study aimed to develop and validate an instrument designed to comprehensively self-assess the acquisition of prehospital disaster response skills during MCI scenario training.

## Methods

### Design

**Figure 1:**
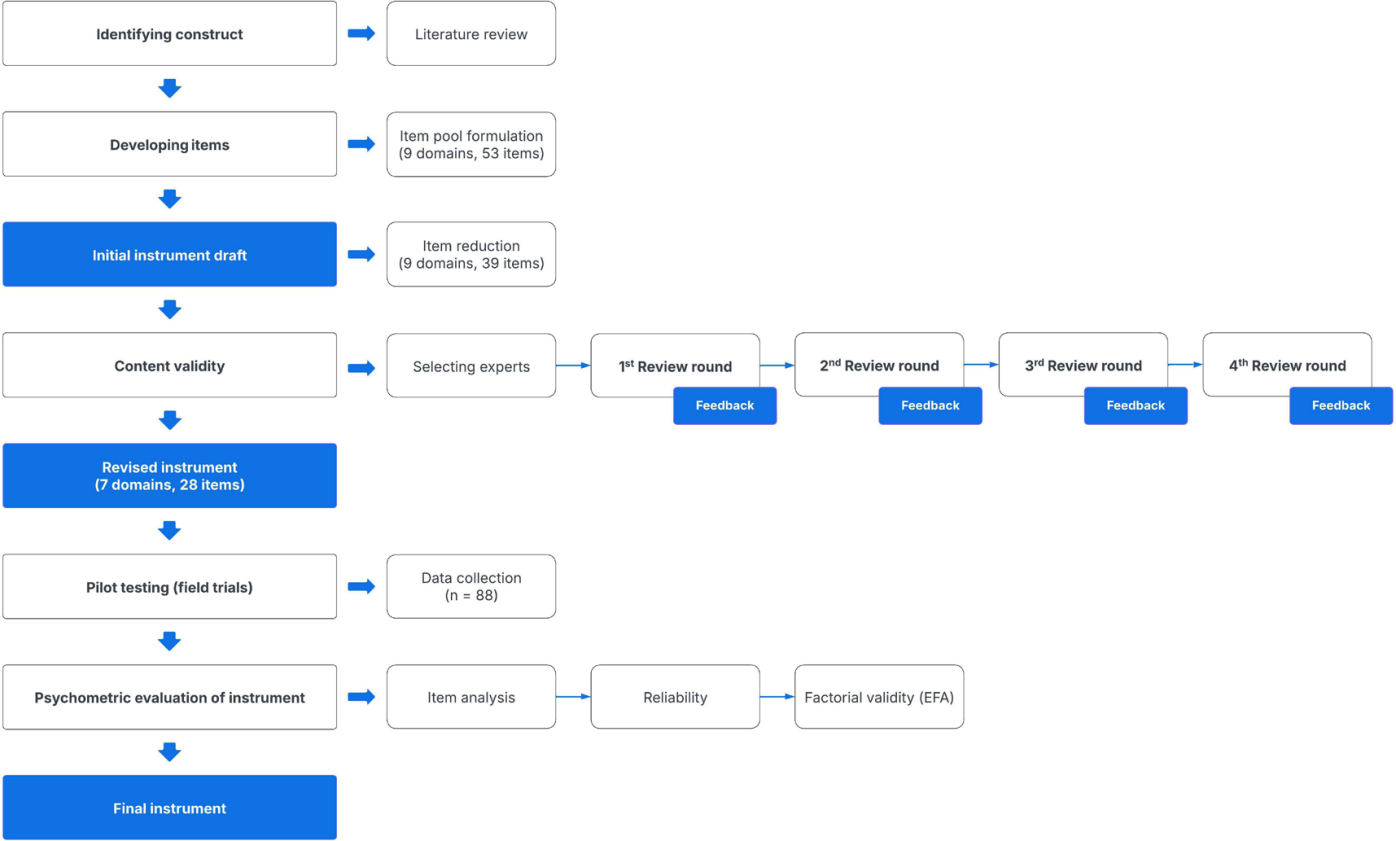
Instrument development and validation process. EFA, exploratory factor analysis.

The development and validation of the instrument followed a systematic approach, adhering to established methodologies for measure development in accordance with the COnsensus-based Standards for the selection of health Measurements INstruments.^12^ This process involved several key steps: identifying the construct, developing the items, ensuring content validity, conducting pilot testing to identify issues and refine the instrument and evaluating the instrument’s psychometric properties.^13^ The process of developing and validating the instrument is illustrated in figure 1.

### Patient and public involvement

Patients and/or the public were not involved in the design, or conduct, or reporting, or dissemination plans of this research.

### Instrument development and validation process

#### Identifying the construct

The first step in the process was to identify and define the construct that the instrument was intended to measure.^13 14^ A comprehensive literature review was conducted to systematically examine and summarise prior prehospital disaster response skills research. This process served both to identify and define the construct while also formulating the item pool. The findings from the literature review were analysed and confirmed through member checking within the research team.^15^ Key themes and concepts were synthesised from the identified literature to generate potential items.^16^ Each item was designed to reflect aspects of the construct, such as risk management, situational awareness and decision-making. This process resulted in an initial pool of 53 items categorised into nine domains. A conservative approach was employed by including more items than anticipated for the final version of the instrument to facilitate subsequent steps of the development process.^13 17^

#### Developing the items

Guided by the model presented by Endsley,^18^ the items were designed and developed to address three knowledge levels. Level 1 assessed whether the training allowed the practice of a specific skill. Level 2 evaluated whether the training led to skill improvement. Level 3 examined whether the training promoted conceptual understanding. Additionally, items assessing the learning process (L) for each identified skill were included in the instrument.

All items used a four-point scale to encourage more definitive responses by eliminating the neutral option, reducing the likelihood of respondents opting for a middle-ground choice.^19^ For the knowledge items numbered 1–21, the scale comprised ‘strongly disagree (1)’, ‘disagree (2)’, ‘agree (3)’ and ‘strongly agree (4)’. For the learning process items numbered 22–28, the scale comprised ‘nothing at all (1)’, ‘little (2)’, ‘some (3)’ and ‘quite a lot (4)’.

The first reduction phase involved removing items that were redundant or not aligned with the study’s aim. This resulted in an initial instrument draft consisting of 39 items categorised in nine domains, which was evaluated for content validity in the subsequent step of the development process.

#### Expert evaluation of content validity

Content validity can be defined as the extent to which an instrument adequately represents the full range of the construct intended to measure.^20 21^ To ensure high content validity for the present instrument, five experts with doctoral degrees and extensive clinical experience in disaster medicine and emergency medical response were individually invited to evaluate the instrument.^21^ They were asked to assess the items for clarity, language complexity and alignment with the defined construct. Each expert provided comments and suggestions for further development in writing. The instrument underwent four rounds of revision, resulting in the final form consisting of 28 items distributed among seven domains: risk assessment, triage, systematic evaluation, situational awareness, mental stress, communication and decision-making. This approach ensured that the final items comprehensively evaluated practical skills, skill development over time and deeper conceptual understanding.

#### Instrument testing

##### Participants

A diverse sample of 75 students (46 women, 28 men and 1 person of undisclosed gender) from a bachelor’s programme at a Swedish university were recruited to participate in the study. The mean age among the participants was 23.8 years (SD: 5.07, range: 20–54). The programme includes comprehensive theoretical and practical training in disaster medicine, equipping students with the skills and knowledge needed to respond to MCIs. This training correlates with the tasks and responsibilities of Swedish EMRs when responding to an MCI.

The bachelor’s programme curriculum covers both technical and non-technical prehospital disaster skills. Technical training includes risk assessment, emergency medical care and the use of specialised equipment. Non-technical training focuses on effective communication, teamwork and decision-making under pressure. The combination of theoretical knowledge and practical application ensures that students are well-prepared to manage the complexities of MCI scenarios, possessing the knowledge and skills expected of healthcare professionals like nurses and physicians in these situations.

##### Pilot testing

Data for the present study were collected through two field trials conducted in May 2023 and May 2024. Each field trial consisted of a 1-week practical exercise featuring a comprehensive training schedule, during which the participants were exposed to a variety of MCI scenarios. The extent of the scenarios ranged from single-vehicle incidents to large-scale events featuring the participation of other first responders. Casualties were portrayed by instructors or fellow participants, guided by emergency medical care instructors with extensive real-world and scenario training experience. After each scenario, the participants received the instrument and provided responses while assessing each item for clarity and alignment with the construct. A total of 88 instruments were gathered during the field trials.

### Data analysis

The data were exported into IBM SPSS Statistics (V.29.0.1.0) for further analysis. The dataset underwent screening for missing values, revealing seven instances in total. Each item was designated with a code (eg, RAS/I) in line with its content and competence level related to the construct. Item analysis and item–total correlation were applied to the data set. This provided an overview of the central tendency and variability of responses, offering insights into the distribution of participants’ self-assessment scores. To determine reliability, both internal consistency and stability were evaluated using Cronbach’s α.^22^

An exploratory factor analysis (EFA) was chosen to evaluate the underlying structure of the data. This allowed for the identification of potential factors without imposing any preconceived hypothesis or models. Furthermore, an EFA provides insight into how well the items measure the intended constructs and whether they align with theoretical expectations.^23^ Kaiser-Meyer-Olkin (KMO) measure of sampling adequacy^24 25^ and Bartlett’s test of sphericity^26^ were implemented to evaluate the suitability of the data for factor analysis. Principal axis factoring was employed as the extraction method. Cases with missing data were excluded listwise, and small coefficients (<0.3) were suppressed to simplify the factor structure. Criteria for determining factor retention included eigenvalues>1, parallel analysis^27^ and the scree plot.^28^ Parallel analysis was conducted using percentile random data eigenvalues (number of datasets=1000, percentage=95). Oblimin rotation with Kaiser normalisation was applied to enhance interpretability.

### Ethical considerations

An application for ethical review of this study was submitted to the Swedish Ethical Review Authority. The study did not involve handling sensitive personal data as defined in §3 of the Ethics Review Act, nor did it include any physical or psychological interventions in humans as specified in §4 of the Ethics Review Act. Consequently, the Swedish Ethical Review Authority waived the need for review (reference: 2021-06241-1). The study adhered to the ethical principles outlined in the Helsinki Declaration. The respondents received written information describing the aim of the study prior to participating in the field exercise. Furthermore, the respondents were informed that their participation was voluntary and that they had the right to withdraw without justification.

## Results

### Item analysis

Item-by-item analysis revealed mean scores ranging from 2.86 to 3.81, with SD between 0.21 and 1.09, indicating moderate response variability. Item–total correlation ranged from 0.29 to 0.55, suggesting that all items had a moderate-to-strong relationship with the total score. The distribution of responses for each item is detailed in table 1.

**Table 1:** Item analysis

Code/competence level*	Item description	Response 1(%)	Response 2 (%)	Response 3 (%)	Response 4 (%)	Missing (%)	Mean/SD
RAS/I	The scenario training gave me the opportunity to practise recognising risks at the scene of an incident.	1.1	10.2	21.6	65.9	1.1	3.57/0.71
RAS/II	I have become better at recognising risks at the scene of an incident.	3.4	10.2	33.0	51.1	2.3	3.40/0.77
RAS/III	I understand more about the importance of recognising risks at the scene of an incident.	5.7	6.8	13.6	71.6	2.3	3.58/0.84
TRI/I	The scenario training gave me the opportunity to practise triage at the scene of an incident.	5.7	13.6	25.0	53.4	2.3	3.27/0.92
TRI/II	I have improved my ability to triage the injured at the scene of an incident.	3.4	19.3	36.4	38.6	2.3	3.12/0.86
TRI/III	I understand more about the importance of following the triage algorithm at the scene of an incident.	3.4	8.0	19.3	67.0	2.3	3.57/0.74
SYS/I	The scenario training gave me the opportunity to practise conducting systematic examinations of the injured at the scene of an incident.	12.5	13.6	23.9	48.9	1.1	3.06/1.09
SYS/II	I have improved my ability to conduct systematic examinations of the injured at the scene of an incident.	6.8	12.5	35.2	43.2	2.3	3.17/0.92
SYS/III	I understand more about the importance of conducting systematic examinations of the injured at the scene of an incident.	3.4	11.4	23.9	60.2	1.1	3.44/0.79
SAW/I	The scenario training gave me the opportunity to practise prioritising information at the scene of an incident.	1.1	10.2	25.0	62.5	1.1	3.51/0.73
SAW/II	I have become better at prioritising information at the scene of an incident.	1.1	13.6	39.8	44.3	1.1	3.32/0.70
SAW/III	I understand more about the importance of being able to prioritise information at the scene of an incident.	–	8.0	28.4	61.4	2.2	3.57/0.63
STR/I	The scenario training gave me the opportunity to practise dealing with mental stress at the scene of an incident.	2.3	5.7	19.3	71.6	1.1	3.63/0.70
STR/II	I have become better at recognising how I react to mental stress at the scene of an incident.	3.4	11.4	28.4	55.7	1.1	3.36/0.83
STR/III	I understand more about the mental stress caused by a mass casualty incident.	1.1	5.7	19.3	72.7	1.1	3.68/0.63
COM/I	The scenario training gave me the opportunity to practise communicating at the scene of an incident.	–	1.1	18.2	78.4	2.3	3.80/0.43
COM/II	I have become better at communicating at the scene of an incident.	3.4	12.5	38.6	45.5	–	3.28/0.81
COM/III	I understand more about the importance of good communication at the scene of an incident.	–	–	4.5	94.3	1.1	3.95/0.21
DEC/I	The scenario training gave me the opportunity to practise making decisions about actions in case of life-threatening conditions at the scene of an incident (eg, stopping catastrophic bleeding).	2.3	11.4	30.7	55.7	–	3.42/0.77
DEC/II	I have become better at making decisions at the scene of an incident.	–	14.8	36.4	48.9	–	3.35/0.71
DEC/III	I understand more about the importance of making quick decisions at the scene of an incident in certain situations.	–	–	20.5	78.4	1.1	3.81/0.39
RAS/L	How much have you learnt today about recognising risks at the scene of an incident?	2.3	15.9	44.3	36.4	1.1	3.19/0.78
TRI/L	How much have you learnt today about triage at the scene of an incident?	8.0	20.5	48.9	21.6	1.1	2.86/0.88
SYS/L	How much have you learnt today about systematic examinations at the scene of an incident?	10.2	18.2	39.8	30.7	1.1	2.88/0.97
SAW/L	How much have you learnt today about situational awareness at the scene of an incident?	1.1	2.3	34.1	60.2	2.3	3.57/0.61
STR/L	How much have you learnt today about dealing with mental stress at the scene of an incident?	2.3	11.4	31.8	52.3	2.3	3.35/0.79
COM/L	How much have you learnt today about communicating at the scene of an incident?	–	5.7	31.8	60.2	2.3	3.56/0.61
DEC/L	How much have you learnt today about decision-making at the scene of an incident?	1.1	17.0	27.3	52.3	2.3	3.35/0.81

* I=opportunity to practice, II=skills improvement, III=conceptual understanding, L=learning process. COM=communication, DEC=decision-making, RAS=risk assessment, SAW=situational awareness, STR=mental stress, SYS=systematic assessment, TRI=triage. Responses for items 1–21: ‘strongly disagree (1)’, ‘disagree (2)’, ‘agree (3)’, ‘strongly agree (4)’. Responses for items 22–28: ‘nothing at all (1)’, ‘little (2)’, ‘some (3)’, ‘quite a lot (4)’.

### Reliability

The reliability analysis revealed an overall Cronbach’s α score of 0.86, indicating high internal consistency. Cronbach’s α scores after deletion of individual items ranged from 0.86 to 0.87, demonstrating that each item contributed positively to the instrument’s overall reliability.

### Factorial validity

The KMO value was 0.704, exceeding the recommended threshold of 0.6. Additionally, Bartlett’s test of sphericity—χ^2^ (378) = 1164.609, p<0.001—was statistically significant, indicating that the correlation matrix was factorable. Measures of sampling adequacy for the 28 items were all above the recommended threshold value of 0.5 (range: 0.53–0.79), which justified the inclusion of all items in the factor analysis.

**Figure 2:**
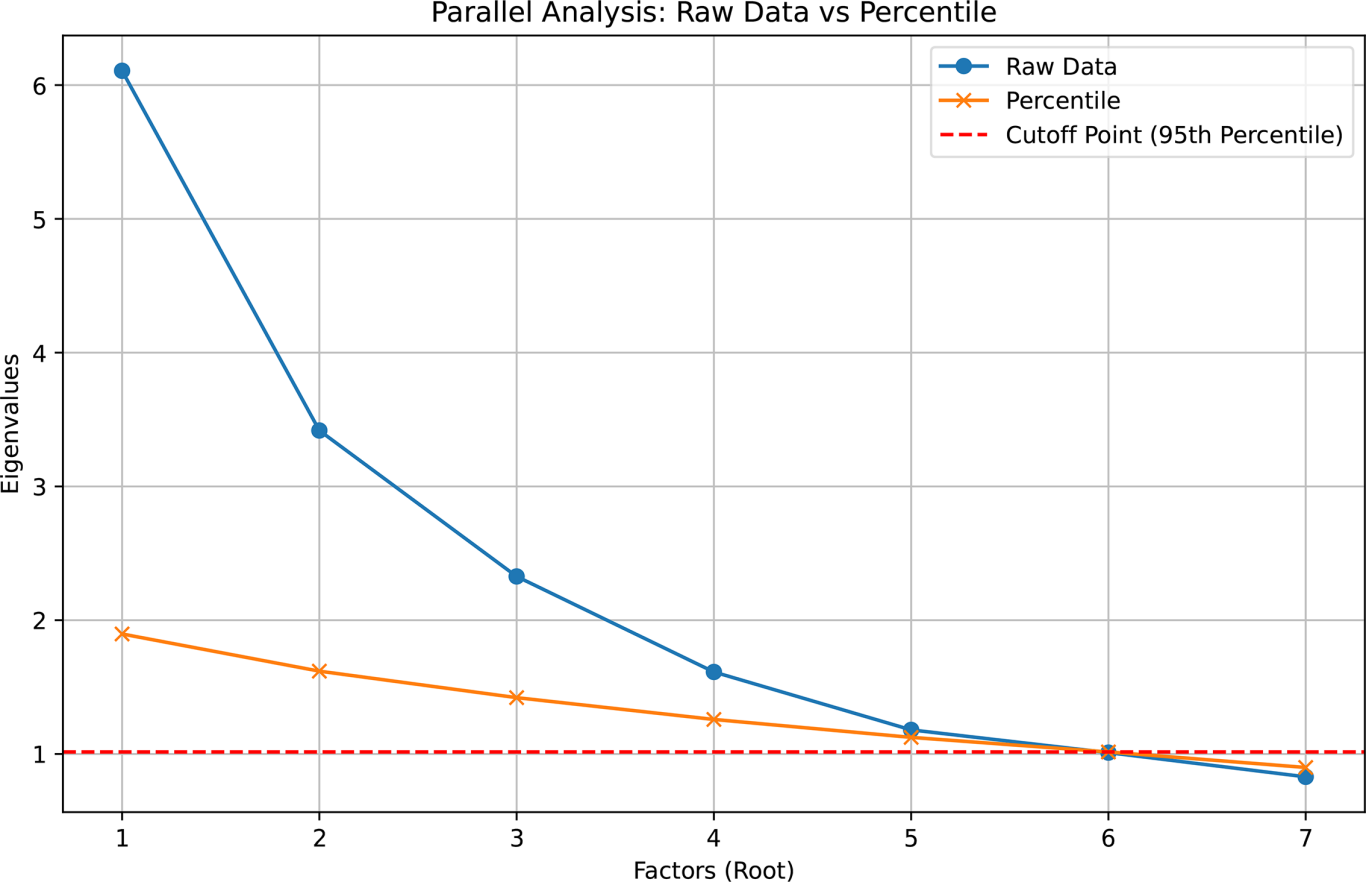
Chart illustrating the differences in factors retained between exploratory factor analysis (blue/circle) and Horn’s parallel analysis (yellow/x).

The EFA initially revealed seven factors with eigenvalues exceeding 1, explaining 23.8%, 13.1%, 9.2%, 6.9%, 5.4%, 4.9% and 4.5% of the total variance, respectively. This was supported by the scree plot, which indicated a breaking point between the seventh and eighth factors. However, Horn’s parallel analysis suggested a cut-off point at five factors compared with the seven factors indicated by the raw data (figure 2). The decision to retain five factors was based on Horn’s parallel analysis, which provided a more stringent criterion. This approach minimised the risk of over-extraction and ensured that only the most robust factors were included, thereby enhancing the interpretability and reliability of the factor solution. The retained five-factor analysis accounted for 52.3% of the total variance. The factors were labelled based on the content of the items that loaded onto each factor: *incident control and management*; *systematic examination procedures*; *risk assessment and management*; *stress response and impact*; and *triage procedures* (table 2). Cronbach’s α for all factors indicated good internal consistency (range: 0.74–0.85).

**Table 2:** Factor loadings of exploratory factor analysis using direct oblimin rotation

Factor labelItem ID/knowledge level*	Factor loading					Explained variance (%)	Cronbach’s α
	1	2	3	4	5	52.3	
Factor 1: incident control and management						21.8	0.84
SAW/I	0.72						
SAW/II	0.71						
DEC/II	0.69						
DEC/I	0.61						
DEC/L	0.57				0.30		
SAW/III	0.54						
DEC/III	0.52						
COM/II	0.48						
COM/I	0.45						
Factor 2: systematic examination procedures						12.2	0.84
SYS/I		0.82					
SYS/L		0.81					
SYS/II		0.69					
SYS/III		0.67					
Factor 3: risk assessment and management						8.3	0.85
RAS/I			−0.85				
RAS/III			−0.84				
RAS/II			−0.80				
RAS/L			−0.71				
SAW/L			−0.36	−0.35			
Factor 4: stress response and impact						5.8	0.75
STR/L				−0.80			
STR/I				−0.72			
STR/III				−0.59			
STR/II				−0.55			
TRI/III				−0.38	−0.34		
COM/L				−0.33			
COM/III				−0.31			
Factor 5: triage procedures						4.2	0.74
TRI/I					−0.58		
TRI/II					−0.57		
TRI/L				−0.32	−0.33		

Note: Principal axis factoring extraction method was used. The rotation converged in 10 iterations.

* I=opportunity to practice, II=skills improvement, III=conceptual understanding, L=learning process. COM=communication, DEC=decision-making, RAS=risk assessment, SAW=situational awareness, STR=mental stress, SYS=systematic assessment, TRI=triage.

## Discussion

This study presents the development and initial validation process of an instrument designed to measure self-assessed learning outcomes in prehospital disaster response skills, using rigorous methodology. The findings indicate that the instrument has overall good to strong psychometric properties with regard to both reliability and factorial validity, suggesting an effective and efficient instrument for use in medical education and simulation training. The discussion is framed within Kane’s framework for validation, which provides a systematic approach to ensure the rigour and credibility of the instrument development process.^29^ By basing our discussion on this framework, we ensure that all necessary steps for developing and validating the instrument have been thoroughly addressed. This includes defining the intended use of the instrument, gathering evidence to support its validity and evaluating the instrument’s reliability. Consequently, using Kane’s framework enhances the robustness of our findings and supports the overall validity of the study.^30 31^

### Scoring

The scoring inference of Kane’s framework pertains to the accuracy and consistency of the scores obtained from the instrument. While the instrument displayed good to strong psychometric properties in terms of reliability and validity, there was an observed ceiling effect, particularly in items related to communication. Previous research identified two main underlying reasons for this effect: the most common being that the previous experience of the participants exceeds the difficulty of the training, thus limiting learning opportunities.^32^

In our study, while the participants received extensive training in the related skills, they were still considered novices compared with professional EMRs. This supports the second previously identified reason for the ceiling effect, namely the correlation between lower levels of experience and an increase in observed ceiling effects.^33^ As indicated by previous literature, altering the number of response options per item could help address the ceiling effect by increasing the granularity of the instrument.^34^ This adjustment could enhance the instrument’s capacity to better accommodate varying skill levels among respondents, from novices to experienced professionals.

### Generalisation

The generalisation inference examines the extent to which the scores can be generalised to the broader population. The results of this study indicate that the instrument demonstrates strong psychometric properties. The reliability analysis, including Cronbach’s alpha (total and if-item-deleted), and the EFA support the internal consistency and factorial validity of the instrument. These findings suggest that the instrument reliably measures the intended constructs and adequately samples the relevant tasks a first responder is expected to perform during an MCI. Therefore, the instrument meets the criteria for consistency of scores and sampling of tasks.

However, the generalisability of the findings is limited by the inclusion of a homogenous group of participants. Despite receiving extensive training, the participants were still novices compared with the education and clinical experience of professional EMRs. This limitation raises concerns about the applicability of the instrument to a broader population. Previous research has highlighted the challenges associated with using homogeneous samples, noting that such samples may not capture the variability present in more diverse populations.^35 36^ Validating the instrument with professional EMRs or international cohorts would provide additional insights into its applicability and ensure that the instrument is robust across different contexts and populations.

### Extrapolation

The extrapolation inference involves the extent to which the instrument’s scores can be used to make valid inferences about the underlying construct of prehospital disaster response skills. Evidence supporting this inference is primarily derived from two sources: methods taken to ensure that the test domain reflects key aspects of real-world performance and empirical analyses evaluating the relationship between test performance and real-world performance.^37^

In the present study, the involvement of experts in disaster medicine and prehospital care ensured that the instrument’s domains accurately reflect the skills required when responding to an MCI. This expert involvement adds credibility to the instrument and supports the validity of the extrapolation inference. While comprehensive empiric analyses are challenging to conduct during the initial development phase, the pilot test provided valuable preliminary data on the instrument’s performance.^38^ Future research should focus on further validating the instrument through extensive testing to strengthen the extrapolation inference.

### Implication

The implication inference considers the practical implications of the instrument’s scores for decision-making and practice. While some instruments aim to evaluate learning outcomes pertaining to skills related to prehospital disaster response, they predominantly focus on a single skill.^39^,^41^ To the extent of our knowledge, the instrument developed and validated for this study is the first that addresses the evaluation of learning outcomes from seven distinct domains: risk assessment, triage, systematic evaluation, situational awareness, mental stress, communication and decision-making. The psychometric properties, including reliability and factorial validity, indicate that the items accurately measure the intended construct. These findings suggest that the instrument is suitable for use in disaster response training programmes.

While the findings of this study support the instrument’s strong psychometric properties, making robust inferences about its practical implications remains challenging.^42^ Our data set is based on pilot tests, which presents context-specific limitations for making strong inferences of the instruments’ real-world implications. In accordance with the present results, previous research has similarly highlighted the difficulties in establishing the practical utility of disaster preparedness tools due to limited real-world data and the need for extensive validation across diverse settings.^43 44^

## Limitations

The instrument relies on self-assessment, which can be subject to biases such as social desirability and self-perception inaccuracies.^45^ Although extensive measures were taken to ensure honest and accurate responses, these biases cannot be completely eliminated. To mitigate these biases and enhance the validity of future studies, integrating objective performance-based measures could be beneficial. Performance-based assessments, which require participants to engage in specific tasks or activities, can complement self-assessment by providing additional insights into participants’ abilities.^46 47^ By combining self-assessment with these objective measures, a more comprehensive evaluation of the instrument’s effectiveness can be achieved.

While the EFA provides strong evidence for the instrument’s structure, it is important to acknowledge that EFA alone has its limitations. EFA is primarily used for identifying potential factor structures without imposing any preconceived constraints on the data. While this is useful for initial exploration, it does not confirm the stability or replicability of the identified factors across different samples or contexts.^48^

Future studies incorporating confirmatory factor analysis (CFA) could further validate the identified factors. CFA would allow for a more rigorous testing of the hypothesised measurement model by specifying the expected relationships between observed variables and their underlying latent constructs. This method would provide additional support for the instrument’s internal structural validity by confirming whether the data fits the proposed model.^49 50^

## Conclusions

This study presents arguments for the validity and reliability of a newly developed instrument for self-assessment of disaster response skills acquired during MCI scenario training. The instrument addresses a critical gap by offering a comprehensive self-evaluation tool that assesses the comprehensive range of skills required for effective disaster response. Its strong psychometric properties in the field test suggest usefulness in similar settings. Future studies should explore the application of this instrument in diverse training settings and among different populations to further enhance its utility and generalisability.

## Data Availability

Data are available on reasonable request.
