# Supplementary material for: Self-assessment of learning outcomes in prehospital disaster response skills: instrument development and validation for mass casualty incident training
Source: BMJ Open. 2025 Mar 27;15(3):e098284. doi: 10.1136/bmjopen-2024-098284 (PMC11956334; doi:10.1136/bmjopen-2024-098284)
Supplement: Supplementary file 1 [file bmjopen-2024-098284.reviewer_comments.pdf]

PEER REVIEW HISTORY

BMJ Open publishes all reviews undertaken for accepted manuscripts. Reviewers are asked to complete a checklist review form and are provided with free text boxes to elaborate on their assessment. These free text comments are reproduced below.

ARTICLE DETAILS

Title (Provisional)

Self-Assessment of Learning Outcomes in Prehospital Disaster Response Skills: Instrument Development and Validation for Mass Casualty Incident Training

Authors

Schulz, Fredrik; Hultin, Magnus; Gyllencreutz, Lina

VERSION 1 - REVIEW

**Reviewer**	**1**
**Name**	**Bijani, Mostafa**
**Affiliation**	**Fasa University of Medical Science, Department of Medical Surgical Nursing, Fasa University of Medical Sciences, Fasa, Iran**
**Date**	**08-Feb-2025**
**COI**	**None**

The topic of the article is very interesting and practical. Although the article is well structured and well written, the following corrections are recommended to strengthen the study: 1- The introduction is based on a structured theoretical framework. Please add a paragraph in the introduction under the title of the pre-hospital emergency situation in Sweden.

2- There are ambiguities in the methodology. What method was used to extract the items? Please provide more details. If a qualitative method was used to extract the items, the type of qualitative study, how the interviews were conducted? and how the qualitative data were analyzed and confirmed(Rigor)should be explained. It is also necessary to explain the validation steps based on the Cosmin checklist(COSMIN (COnsensus-based Standards for the selection of health status Measurement INstruments) checklist for evaluating the methodological quality of studies on measurement properties.

Was item analysis performed before conducting the exploratory factor analysis? And if not, what was the reason?

Why was confirmatory factor analysis not used?

The discussion is very poorly written and needs strengthening.

**Reviewer**	**2**
**Name**	**Goniewicz, Krzysztof**
**Affiliation**	**Polish Air Force Academy**
**Date**	**10-Feb-2025**
**COI**	**None**

How might the response scale be adjusted to better differentiate skill levels, particularly among more experienced professionals?

The generalizability of the findings is limited by the use of a student sample from a single university. Would validating the instrument with professional emergency responders or international cohorts provide additional insight into its applicability?

The EFA provides strong evidence for the instrument’s structure. Could a future study incorporating CFA further validate the identified factors?

How might this tool be adapted for use in real-world disaster response training programs or professional development courses?

The limitations section already acknowledges the reliance on self-assessment, which may introduce biases such as social desirability. Would integrating an objective performance-based measure in future studies improve the validity of the findings?

Consider : https://doi.org/10.1186/s12889-021-10165-5

VERSION 1 - AUTHOR RESPONSE

Reviewer #1

Comment	Note(s)
The introduction is based on a structured theoretical framework. Please add a paragraph in the introduction under the title of the pre-hospital emergency situation in Sweden.	We have made corrections under the subheading Participants to better present the Swedish context of the study.<br><br>While we have not structured the introduction around a theoretical framework, we hope these changes provide a clearer understanding of the participants' background and the relevance of their training to the study.
There are ambiguities in the methodology. What method was used to extract the items? Please provide more details. If a qualitative	We have revised the methodology section to provide more details on the item extraction process.

method was used to extract the items, the type of qualitative study, how the interviews were conducted? and how the qualitative data were analyzed and confirmed (Rigor) should be explained.	Specifically, we have clarified the steps taken to synthesize key themes and concepts from the literature.<br><br>Additionally, we have outlined how member checking within the research team was employed to analyse and confirm the findings, ensuring rigor. These revisions aim to address the ambiguities and provide a clearer understanding of the methods used.
It is also necessary to explain the validation steps based on the Cosmin checklist (COnsensus-based Standards for the selection of health status Measurement INstruments) checklist for evaluating the methodological quality of studies on measurement properties.	We have revised the text to outline the implementation of the COSMIN checklist throughout the development and validation process where applicable. Additionally, Figure 1 has been added to illustrate these steps, providing a comprehensive overview of the process and ensuring clarity in the validation steps taken.
Was item analysis performed before conducting the exploratory factor analysis? And if not, what was the reason?	We have corrected the subheading to better reflect the item analysis conducted.<br><br>Additionally, Table 1 has been relocated from the Methods section to the Results section. We hope these changes enhance the presentation of the item analysis and subsequent findings.
Why was confirmatory factor analysis not used?	We have revised the text to more accurately explain the rationale for choosing an Exploratory Factor Analysis (EFA) for the present study.<br><br>Additionally, we have acknowledged the value of conducting further studies with a Confirmatory Factor Analysis (CFA) in the Limitations section.
The discussion is very poorly written and needs strengthening.	We have significantly revised the Discussion section to enhance its clarity and depth. This includes incorporating additional citations to strengthen our arguments and provide a more robust analysis. Additionally, we have expanded the Limitations section to further support the discussion and address potential concerns.

Reviewer #2

**Comment**	**Note(s)**
How might the response scale be adjusted to better differentiate skill levels, particularly among more experienced professionals?	Yes, the response scale did not differentiate experienced professionals regarding skills levels in this cohort.<br><br>The revised text provides a more detailed explanation of the observed ceiling effect and suggests practical adjustments to the response scale to enhance the instrument’s capacity to accommodate varying skill

	levels among respondents, from novices to experienced professionals.<br><br>Additional citations have been included to further substantiate the text.
The generalizability of the findings is limited by the use of a student sample from a single university. Would validating the instrument with professional emergency responders or international cohorts provide additional insight into its applicability?	The revised text acknowledges the limitations of using a homogenous student sample and emphasizes the need for further validation with professional emergency responders and international cohorts. This approach will provide additional insight into its applicability and ensure its robustness across different contexts and populations. We have also included additional citations to support these points.
The EFA provides strong evidence for the instrument's structure. Could a future study incorporating CFA further validate the identified factors?	We have expanded the Limitations section to acknowledge the limitations of using EFA alone and to emphasize the importance of future studies incorporating Confirmatory Factor Analysis (CFA). This addition highlights that while EFA provides strong evidence for the instrument's structure, CFA would offer a more rigorous testing of the hypothesized measurement model and further validate the identified factors.<br><br>We believe this expansion addresses your concern and strengthens the manuscript.
How might this tool be adapted for use in real-world disaster response training programs or professional development courses?	We have expanded the Discussion section to address this issue. The revised text highlights the instrument's suitability for disaster response training programs by emphasizing its comprehensive evaluation of learning outcomes across seven distinct domains.<br><br>Additionally, we acknowledge the challenges of making robust inferences about the instrument's practical implications based on pilot study data and emphasize the need for further validation with diverse populations. We have also included additional citations to support these points. This approach will provide additional insight into its applicability and ensure its robustness across different contexts and populations.
The limitations section already acknowledges the reliance on self-assessment, which may introduce biases such as social desirability. Would integrating an objective performance-based measure in future studies improve the validity of the findings?	We have revised the Limitations section to address this issue. The revised text acknowledges the limitations of self-assessment and suggests that integrating objective performance-based measures in future studies could enhance the validity of the findings. We have also included additional citations to support these points.

VERSION 2 - REVIEW

Reviewer 1

Name Bijani, Mostafa

Affiliation Fasa University of Medical Science, Department of Medical Surgical Nursing, Fasa University of Medical Sciences, Fasa, Iran

Date 18-Mar-2025

COI

Considering that the author has made the corrections and the article has been improved, the article is worthy of publication.
